# A facile and low-cost micro fabrication material: flash foam

**DOI:** 10.1038/srep13522

**Published:** 2015-08-28

**Authors:** Yong He, Xiao Xiao, Yan Wu, Jian-zhong Fu

**Affiliations:** 1 The State Key Lab of Fluid Power Transmission and Control, College of Mechanical Engineering, Zhejiang University, Hangzhou 310027, China; 2 Key Laboratory of 3D Printing Process and Equipment of Zhejiang Province, College of Mechanical Engineering, Zhejiang University, Hangzhou 310027, China

## Abstract

Although many microfabrication methods have been reported, the preliminary replication templates used in most microfabrication still depend on the expensive and long-period photolithography. This paper explores an alternative replication templates based on a daily used material, flash foam (FF), and proposes a facile microfabrication method, flash foam stamp lithography (FFSL). When FF is exposed with a desired pattern mask, the negative of the pattern is transferred to its surface and micro structures are formed due to the shrinkage of the exposed area. As FF is commonly used in personal stamps, FFSL is very simple and cost-effective. In this paper, we demonstrated that FF is a good and low-cost template for many micro fabrication methods, such as micro casting and soft lithography. Thus, designing and fabricating micro structures at personal office immediately become possible with FFSL. Furthermore, we demonstrated that multi-scale micro structures can be easily fabricated by double exposure with FFSL. Skin textures is used as another case to demonstrate that FFSL can fabricate structures with different depth in a single exposure. As a result, FF shows a promising future in biology, and analytical chemistry, such as rapid fabrication of point of care diagnostics and microfluidic analytical devices with low cost.

As the primary supporting technology for micro-electromechanical systems (MEMS), fabrication of desired micro structures has attracted much interest from researchers and developers in recent decades. A variety of micro fabrication methods have been explored, including photolithography^1^,^2^, chemical etching^3^,^4^, LIGA^5^,^6^, micro hot embossing (μHE)^7^,^8^, nanoimprinting^9^, soft lithography/micro contact printing (μCP)^10^,^11^, micro injection molding^12^,^13^, and micro machining^14^,^15^ *et al.* Some methods are physical processes, for example, μHE; some are chemical processes, like chemical etching, and some are specially designed for fabricating polymer, such as micro injection molding. Each method is advantageous for certain specific applications; although certain limitations still exist. Many of these micro fabrication methods require specialized, expensive, or complicated equipment, which make fabrication become costly and only be suitable for mass production. Photolithography, LIGA, micro injection molding, and micro machining fall into this category. To reduce the cost of LIGA, UV-LIGA was developed in recent years by avoiding the use of synchrotron radiation X-ray lithography^16^. However, as photolithography is also needed in UV-LIGA, this fabrication method still can’t be treated as low cost. μHE is often used to imprint micro structures and to achieve many success in mass fabrication; however, the mold used for imprinting/embossing is expensive, which prevents its wider application in prototyping research. Typical micro fabrication with chemical processes requires toxic intermediate materials which must be handled by specially-trained staff; as a result, microfabrication with chemical processes such as chemical etching still needs to be improved.

Soft lithography represents a stamping fabrication method for rapid prototyping of various types of both microscale and nanoscale structures, applicable to devices on planar, curved, flexible, or soft substrates especially when low cost is required^17^. The two primary considerations of this method includes the fabrication of an elastomeric stamp, and transferring the pattern from the stamp to the substrate. Elastomeric stamps, made of poly (dimethyl siloxane) (PDMS), are typically casted from photolithography templates, which are very costly, though soft lithography is cheap overall. The stamping process requires careful handling, as PDMS does not absorb ink and ink thickness on the stamp surface is difficult to control^18^. If the stamp could be directly fabricated and could absorb ink, soft lithography will be more facile.

In general, a micro replication template or mold is necessary in almost every widely used microfabrication method, which is mainly fabricated by photolithography. In many cases, researchers want their ideas to be realized as soon as possible and to avert expensive cost due to the repetitive revisions in the following experiments. To meet these requirements, a flexible and economical microfabrication method is proposed to avoid the high-cost and long-term fabrication period of micro mold.

Flash foam (FF), also called photosensitive seal, is a type of ultra-micro bubble material, typically made of polyethylene, which has been invented since 1990s and designed for fabrication of personal stamp. The most unique characteristic of FF is that when it is exposed to an intense burst of light, its micro-porous surface will be sealed – a mask on top of the flash foam is exposed, transferring the pattern to the flash foam to create a flash foam stamp (FFS). Ink is stored in the porous FF and then stamped onto paper through the unsealed surface area. Thus, flash foam stamps are more convenient than traditional hard stamps due to their high resolution and inkpad-free stamping process. Currently, FFS is already widely used to fabricate many types of stamps and cost less than $0.2/each. The fabrication cost is basically negligible compared to other methods if it can be used in micro fabrication. Although FF has achieved big success in our daily life, discussions about applications of this material in fabricating micro structures have never been reported as far as we know.

This paper discusses the potential applications of FF in microfabrication and proposes a novel microfabrication method called flash foam stamp lithography (FFSL). FF is already quite commonly used, and is cheap and simple to make, which leads to the potential application as a flexible, affordable and facial micro fabrication method for even wider use, especially in soft lithography field. Furthermore, we demonstrate two unique properties of FFSL including fabricating multi-scale micro structures with double-exposure and forming micro structures of different depth with single-exposure.

## Fabrication of FFS

**Figure 1:**
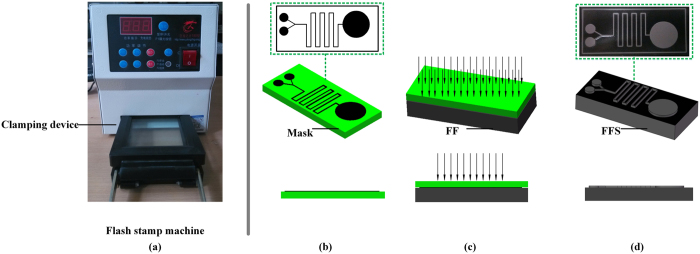


The FFS machine as shown in Fig. 1a was purchased from Liaocheng Beike Electronic Information Materials Co., Ltd. (Liaocheng, China). Fabrication process of FFS is very simple, as shown in Fig. 1b-d. First, the desired patterns were designed using CAD software (CorelDraw or AutoCAD *etc.*) then printed onto the tracing paper with an inkjet printer to obtain the mask as shown in Fig. 1b. The resolution of mask directly determines the resolution of the FFS, so high resolution in printing is preferable. If the printed mask is still below the resolution standard, photo mask (chrome mask, emulsion mask and film mask) commonly used in photolithography can be adopted instead. Both the mask and the flash form are pressed together by a clamping device; then exposed in the flash stamp machine with Xenon tubes. Thus, inside the machine, the intense burst of light will seal the non-printing area of the mask (Fig. 1c), finally achieve the FFS with micro structures, as shown in Fig. 1d.

**Figure 2:**
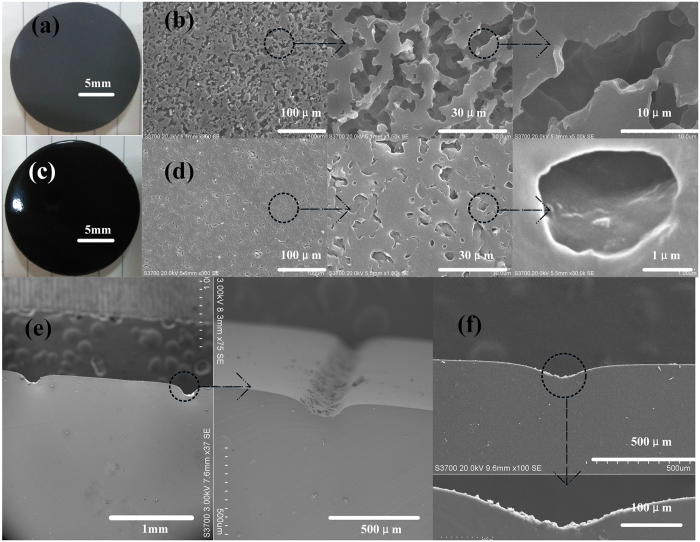
SEM of the FFS cross section, (**a**) and (b) before exposure, (c) and (d) after exposure with a mask, (e,f) micro channel caused by shrinkage of exposure area. (**e**,**f**) are the PDMS replications of FF.

**Figure 3:**
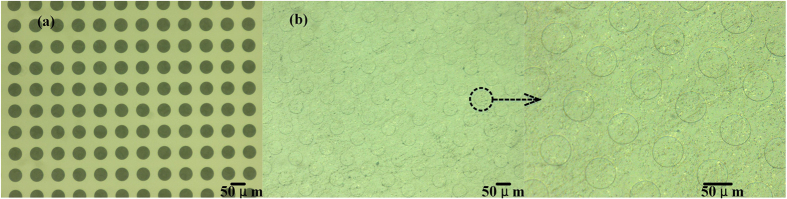
Micro structure on the FFS, (a) chrome mask, (b) after exposure, pattern transferred from mask to FFS. First of all, FF was exposed 3 times without mask to make the surface smooth (Sealing the micro pores shown in Fig. 2b). Then, FF with chrome mask was used in exposure with 7 times to transfer circle to the FFS.

As shown in Fig. 2a,b, FF is a microporous material with an average diameter less than 30μm which can store oils in its pores. Once exposed to bright light, the FF will absorb the energy and the exposure area will melt. After exposure, the temperature will fall rapidly and the exposed area of the FF will form a film, which seals the porous material and isolates it from ink. (This is why an FFS is also called a “photosensitive seal stamp”.) As shown in Fig. 2c,d, the micropores shrink and close after exposure, from unsealed size of 20–30 μm to sealed size of 2-3 μm, to avoid ink passing through the area. Due to the shrinkage of the exposed area, a concave pattern can be acquired, as shown in Fig. 2e,f. A typical micro structure with a size of 50 μm on the FFS is shown in Fig. 3, where the mask pattern is completely transferred to the FFS. The entire process only requires a cheap FFS machine (about $100,) instead of the expensive photolithography machine and/or toxic materials needed for other methods.

## Micro Fabrication Based on FFSL

### Functional structures fabrication

**Figure 4:**
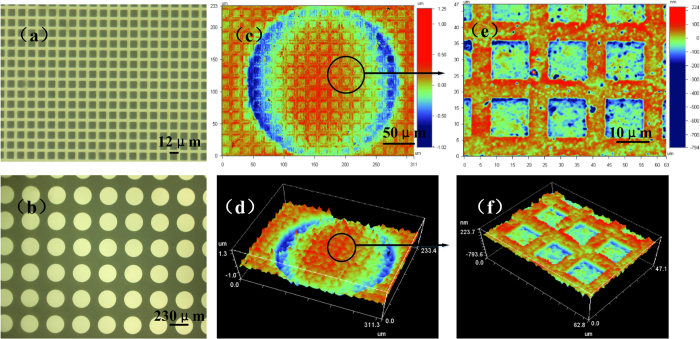
Multi-scale structures fabricated by FFSL. (**a**) The small size of chrome mask with a serial of 12 μm squares which can be directly penetrated by light. (**b**) The large size of chrome mask with a serial of 230 μm circles, which cannot be penetrated by light. (**c**,**d**) Fine products. (**a**,**b**) were measured by a digital microscope (SRT6200, Keyence) and (**c**–**f**) were measured by an optical profiler (Wyko NT9100, Veeco). First FF was exposed 3 times without mask to make the surface smooth (Sealing the micro pores shown in Fig. 2b). Then FF with small size mask was used in exposure with 7 times to transfer rectangle to the FFS. At last circle mask was used in exposure with 2 times.

Multi-scale structures is usually found in nature, for example the foot hair of gecko^19^ and the leaf surface^20^. Recently, many fabrication methods have been developed to fabricate multi-scale structures, such as electrohydrodynamic^21^,^22^ and solution casting^23^. In this paper, we demonstrated that through FFSL this kind of structure can be rapidly fabricated based on two-step-exposure method. Firstly, the small size structure is transferred to FF after exposure; then the FFS with small structure is exposed under the mask with large size structure. Thus, a multi-scale structure with large convex cylinders and small concave squares can be achieved, as shown in Fig. 4. Through changing the pattern of mask, the structure with convex-large-scale and convex-small-scale can also be fabricated, as shown in Fig. S1.

**Figure 5:**
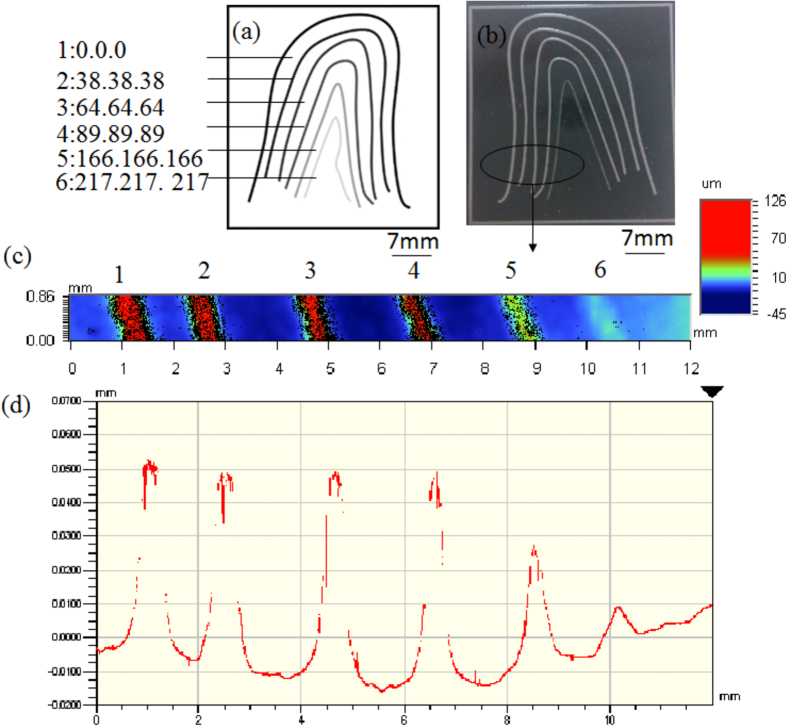
Different depth fabricated with a single exposure. (**a**) The mask printed with a different grayscale. (**b**) FFS fabricated with this mask. (**c,d**) Six lines with different height (depth), measured by an optical profiler (Wyko NT9100, Veeco).

**Figure 6:**
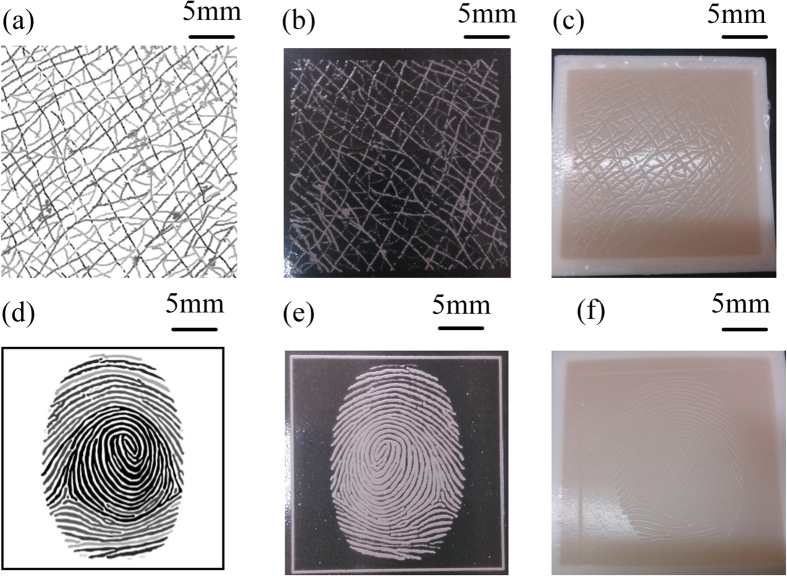
Skin texture. (**a,d**) mask, (**b,e**) FFS, (**c,f**) silicon skin fabricated by micro casting with FFS template.

Fabrication of skin texture is also very attractive, however it is difficult to mimic the depth variation in a real skin with current fabrication method. In experiments, we found that FF has an interesting feature that the depth of the exposure area is influenced by the light intensity. Thus, if a mask is printed with different grayscale, the different height/depth on the FFS can be achieved after exposure. As shown in Fig. 5a, six lines with same width but different grayscales from RGB (0, 0, 0) to RGB (217, 217, 217) were designed on a mask. After exposure, six lines with different heights, from 50 μm to 9 μm, were acquired (Fig. 5b–d). With FFSL, we can build a table which contains the relationship between the grayscale and the height/depth. With this table, we can fabricate a totally 3D micro structure with a 2D mask. As shown in Fig. 6, more realistic skin texture including the fingerprint can be easily fabricated through FFSL.

### FFS as the mold for casting

Though many micro replication methods, such as μHE and micro casting, seem to be cost-effective, the mold used for replication is much more expensive which increases the overall cost for the prototyping fabrication in research. In effort to remedy this problem, this study develops a micro casting method with lower cost based on FFS.

**Figure 7:**
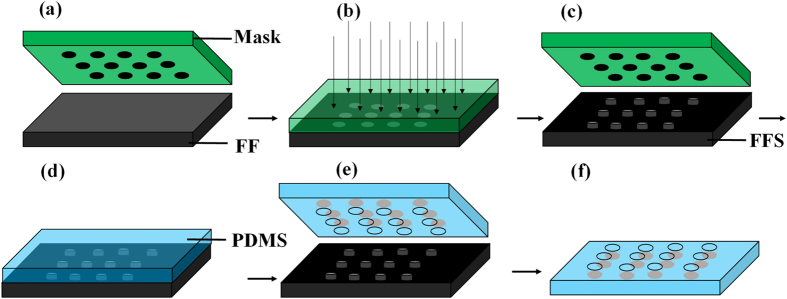
Micro casting process with FFS mold. Fabrication of FFS, (**a–c**). Casting PDMS, (**d**). PDMS removal to acquire fine micro structure, (**e**,**f**).

**Figure 8:**
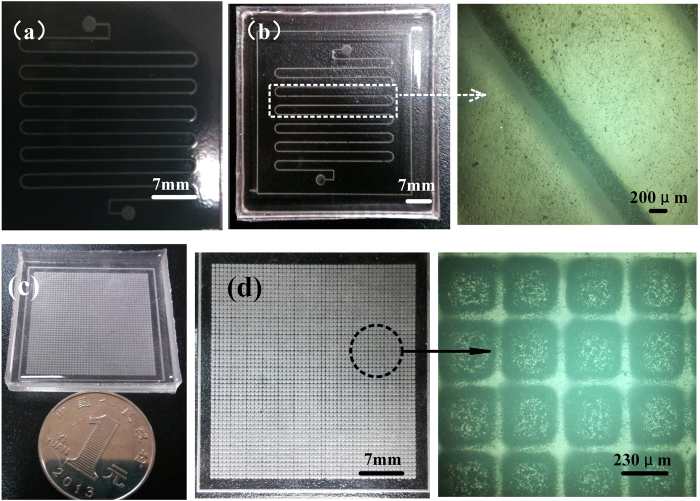
Micro casting based on FFS. (**a**) FFS mold. (**b**) PDMS micro channels with micro casting. (**c,d**) Rectangle array of PDMS fabricated by FFSL. The micrographs were measured by a digital microscope (SRT6200, Keyence). To make sure the FFS is clean, it was first dipped in ethanol for 5 min then ultrasonically cleaned in deionized water for 10min. Silicon oil was spin-coated on the FFS in order to peel off the resultant PDMS mold more easily. PDMS is a very attractive polymeric material for the manufacture of microfluidic channels, due to its favorable optical properties, non-toxicity, and ease of bonding. This study used Sylgard 184 (Dow corning, MI, USA), specifically, which was stirred thoroughly with curing agent at a weight ratio of 10:1. The prepared PDMS mixtures were first placed in a vacuum desiccator for 30 min for degassing, and then poured onto the photosensitive stamp, followed by vacuum degassing for 30 min. The stamp was then placed in an oven to bake at 65 °C for 2 h. After cooling to room temperature, the PDMS sheets were carefully peeled off from the mold and cut into appropriate size. The PDMS was then rinsed in ethanol for 5 min and ultrasonically washed in deionized water for 30 min to sterilize and clean thoroughly.

As shown in Fig. 7, the process used in this study only has two steps: fabrication of the FFS, and casting. Because of the shrinkage at the exposure area, the micro structure on the FFS has an appropriate height and is an ideal mold for micro casting. Micro channels, the main structures of a microfluidic chip, and a micro rectangle array were cast from the FFS stamp as shown in Fig. 8. Comparatively speaking, traditional methods waste much time in fabricating the casting mold, (typically, 2-3 days are needed to create a mold,) and the whole process is also costly. However, using FFSL, the structure such as microfluidic chip and micro array can be designed and fabricated immediately for testing, which saves much time at the prototyping stage.

**Figure 9:**
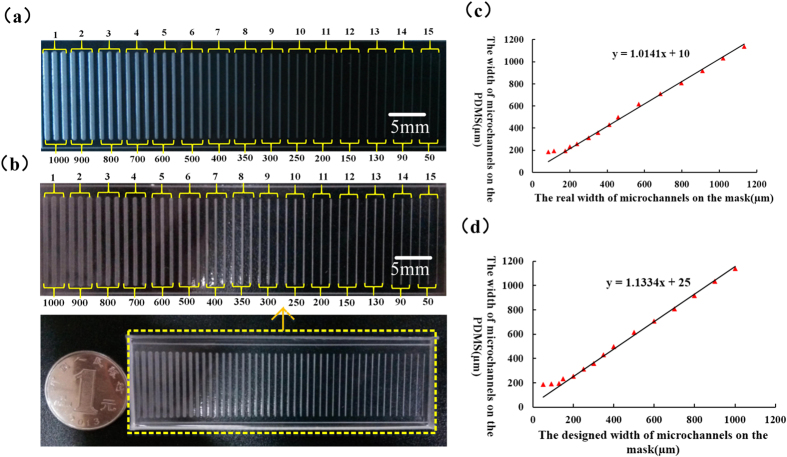


To determine the replication resolution of the FFSL method, the final widths of micro channels were studied in the range of 50–1000 μm with designed widths of 50 μm, 90 μm…1000 μm, as shown in Fig. 9a. The PDMS replication is shown in Fig. 9b. Each channel was repeated three times in the mask. The primary goal here is to demonstrate that FFSL can be used in the common laboratory to fabricate micro structures. The mask was printed on a trace paper by HP laser printer with a printing resolution of 600DPI. Theoretically, the minimum diameter of each ink droplet of a printer with 600DPI is about 42.3 μm, which means that the smaller line sizes cannot be printed precisely. For example, the designed line width on the mask as shown above was 90 μm, while the actual size on the mask was 125 μm. The relationship between mask size and replication size is detailed in Fig. 8c,d. Size error occurs when the pattern is transferred from the mask to the final replication, but the error is linear. As a result, it is still easy to acquire desired size (Fig. 9c,d), especially when the dimension of the structure is larger than 200 μm. Generally, structures within this dimension range can be easily fabricated in any common office environment. As far as cell culture, this size is most useful for cell reproduction. As printing the mask film is very cheap and easy with the commercial inkjet/laserjet printers (Konica Minolta C353, Epson 1390 and Fuji Xerox C800). A serial supplemented experiments are used to quantify the resolution of mask films, FFS molds and the replication micro structures. As shown in Fig. S2, almost each commercial printer can well print mask film and there is no significant difference of the FFSL resolution with different printers. In most case, we can use the linear relationship of designed sizes and the replication sizes to predict the sizes of replication structures.

**Figure 10:**
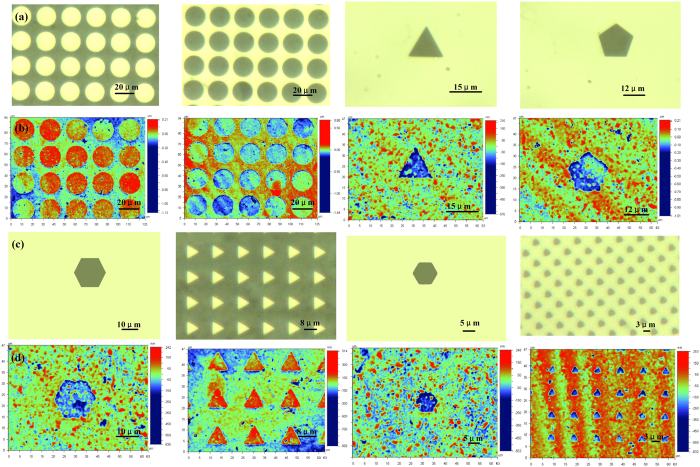
Resolution of FF; (a,c) chrome mask, (b,d) micro structures on the FF with the corresponding mask. (**a,c**) were measured by a digital microscope (SRT6200, Keyence) and (**b,d**) were measured by an optical profiler (Wyko NT9100, Veeco).

To test the final resolution that FF can achieve, a serial of photo masks (chrome mask) sizes include 20 μm, 15 μm, 12 μm, 10 μm, 8 μm, 5 μm and 3 μm were used to fabricate FFS (Fig. 10a,c). It can be found that when the pattern size on the mask is large than 10 μm, it can be well transferred to the FF with a size error less than 1 μm, which was shown in Fig. 10b,d. However when the mask size is below 5 μm, the structures can’t be transferred well. As we mentioned, the micropores on the FF shrink after exposure, from unsealed size of 20–30 μm to sealed size of 2-3 μm (Fig. 2). So the fine resolution of FFSL is about 2-3 μm, that is the reason why the size of 5 μm can’t be fabricated well, which means FFSL may be not suitable for the nanofabrication. When the micro structures are less than 100 μm, chrome mask usually used in photolithography is recommended as printed mask films have low resolution at that scale.

### FFS stamps for soft lithography

Soft lithography is a powerful research tool for biologists and chemists. The stamp type most commonly used in soft lithography is PDMS, fabricated by micro casting. Its casting mold is made by photolithography, which is highly expensive. Additionally, the stamp pattern usually requires modification, which is time-consuming and expensive. If the stamps are more easily to be fabricated, soft lithography will be more feasible and lightweight.

In this study, FFSL’s application in soft lithography for fabrication of microfluidic paper-based analytical devices (μPADs) is demonstrated. μPADs, first proposed by Martinez *et al.*^24^, have received plenty of attention from researchers due to their favorable potential application in disease diagnosis and biochemical analysis^25^,^26^. They possess many attractive features including usability, low cost, low consumption of reagents and samples, pumpless driving, portability, and disposability. The key component of μPAD fabrication is the construction of hydrophobic barriers. Many fabrication methods have been proposed to fabricate the barriers; however, a lightweight μPAD fabrication method with sufficient middle resolution, low cost, and easy implementation is yet underdeveloped.

**Figure 11:**
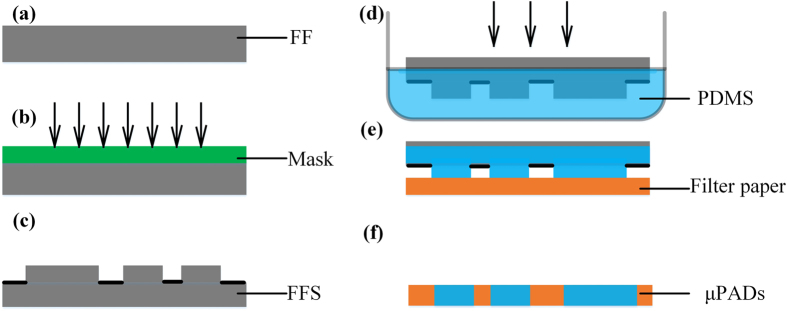
Stamping μPADs with FFS. Fabrication of FFS, (**a–c**). Absorbing PDMS ink, (**d**). Stamping on filter paper, (**e**). After PDMS solidified, patternized hydrophobic barriers are acquired and μPADs are formed, (**f**).

As shown in Fig. 11, the fabrication process with FFSL is very simple. It only requires fabrication of the FFS, (Fig. 11a–c) and stamping, (Fig. 11d–f). First, an FFS with designed channels was fabricated, then immersed in hydrophobic solvent to absorb ink for about 15 min. When the FFS stamped on the paper, the hydrophobic solvent was transferred to the paper. μPADs with hydrophobic barriers were obtained after the hydrophobic solvent solidified either in a vacuum oven at 60 °C for 15 min, or at room temperature for an hour. PDMS was used here for stamping ink, as it is widely used in analytical chemistry and displays favorable characteristics. (A detailed discussion on use of FFSL to fabricate μPADs can be found in our recent publication^27^).

### Cost analysis

**The primary advantages of FFSL are low cost and convenience. A commercial FFS machine **only costs about $100–$300, and has portable and compact size of about 320 mm × 170 mm × 170 mm and weight of 10 Kg. The mask used in FFS can be printed with a very common inkjet or laser printer, which makes it possible to do some researches at office. The main material, flash foam, is also very cheap at about $10/m^2^–$100/m^2^. The material cost is about $0.02–$0.2 for a common stamp sized 40 × 40 mm^2^, which can be essentially negligible. Micro structures above the sizes of 200 μm could be precisely fabricated using the mask printed with a common laser inkjet printer (600DPI). Now the commercial level inkjet printers can achieve a resolution of 2400DPI, so designing and fabricating micro structures above 50 μm immediately can be expected in everyone’s office with a low cost.

## Discussion

FFS fabrication is very quick – two to ten times exposure only take about 3–5 min. It is a facile and lightweight method which presents the potential application in many relevant fields. Use of FFS is rather attractive for applications that require templates. As flash foam is soft enough, it is more suited to the micro fabrication of structures with appropriate mass area and low aspect ratio. Further application of FFSL is still pending, especially as far as its use in micro devices for biology, medicine, or analytical chemistry, including fabrication of organs on chips, micro total analytical systems (μTAS), and others.

Two unique characters of FFSL including easily fabricating multi-scale structure with two step exposure and micro structures with different heights at one step exposure were also demonstrated. Theoretically, real 3D micro structure can be fabricated by controlling the grayscale distribution on the mask. Although the concept of MEMS has been proposed for more than a decade, MEMS is still not as successful as it was expected. One reason is that an initial replication template of almost every microfabrication method must be fabricated by photolithography. The expensive cost and long fabrication cycle in prototyping stage restrict the improvement of MEMS products. In our research, we not only require that “what you see is what you get”, but also require that “what you see becomes what you get as soon as possible”. The core question is that can we design a micro device and fabricate it within an hour? In this paper, we demonstrated that FFSL can be used to fabricate the replication template rapidly and cost effectively. The researchers in biology, medicine, analytical chemistry can use this facile method to accelerate their research.

## Conclusion

Currently, low-cost micro fabrication methods, such as micro casting or μHE, are only cheap at the mass fabrication level, not at prototyping one. These methods typically require expensive molds as replication templates. In most cases, however, prototyping micro devices is only needed for experiments. This study proposes a novel micro fabrication method to solve this problem. FFSL can directly fabricate micro molds or micro stamps at an incredibly low cost. It is a universal micro fabrication method suitable for researchers in practically any laboratory. Applications based on FFSL were also discussed here, and FFSL was successfully used for micro casting and soft lithography. A 200 μm micro structure was able to be fabricated easily in a common office environment. The proposed method has promising potential application in the biology field, as many cells tend to grow at the same size as the sample developed in this study.

## Additional Information

**How to cite this article**: He, Y. *et al.* A facile and low-cost micro fabrication material: flash foam. *Sci. Rep.* **5**, 13522; doi: 10.1038/srep13522 (2015).

## Supplementary Material

Supplementary Information
