# Supplementary material for: A facile and low-cost micro fabrication material: flash foam
Source: Sci Rep. 2015 Aug 28;5:13522. doi: 10.1038/srep13522 (PMC4551987; doi:10.1038/srep13522)
Supplement: Supplementary Information [file srep13522-s1.doc]

**A facile and low-cost micro fabrication material: flash foam**

Yong He^1,2^*, Xiao Xiao^1,2^, Yan Wu^1,2^, Jian-zhong Fu^1,2^

*(1. The State Key Lab of Fluid Power Transmission and Control, College of Mechanical Engineering,*

*Zhejiang University, Hangzhou 310027, China*

*2. Key Laboratory of 3D Printing Process and Equipment of Zhejiang Province, College of Mechanical Engineering, Zhejiang University, Hangzhou 310027, China*

**Correspondence to: Jian-zhong Fu; e-mail:* [*fjz@zju.edu.cn*](mailto:fjz@zju.edu.cn))

Supplementary Materials


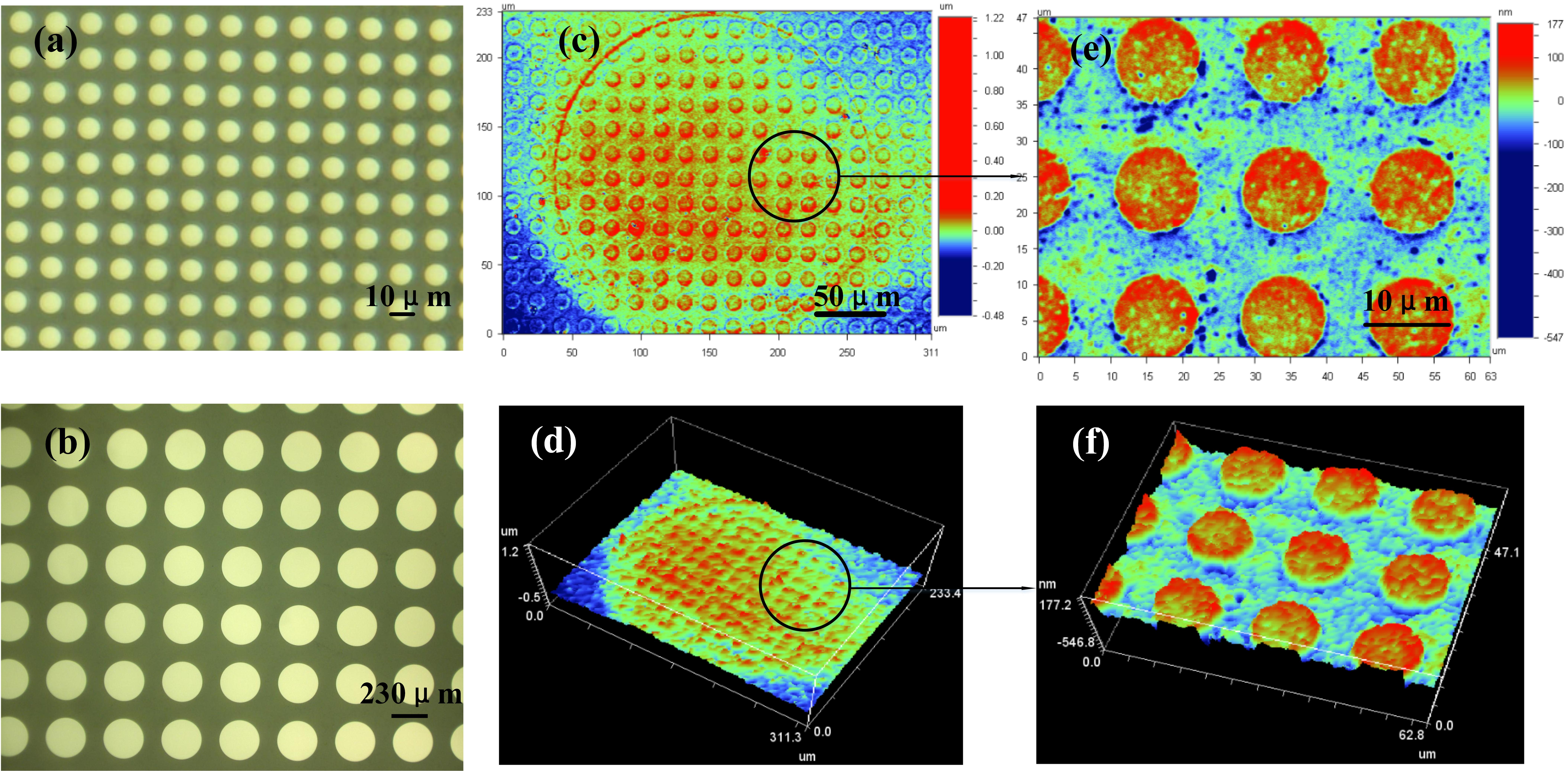


Fig. S1: Multi-scale structures fabricated by FFSL. (a) The small size of chrome mask, 12μm circle. (b) The large size of chrome mask, 230μm circle. (c) & (d) Fine products. (a) & (b) measured by a digital microscope (SRT6200, Keyence), (c), (d), (e) &(f) measured by an optical profiler (Wyko NT9100, Veeco).


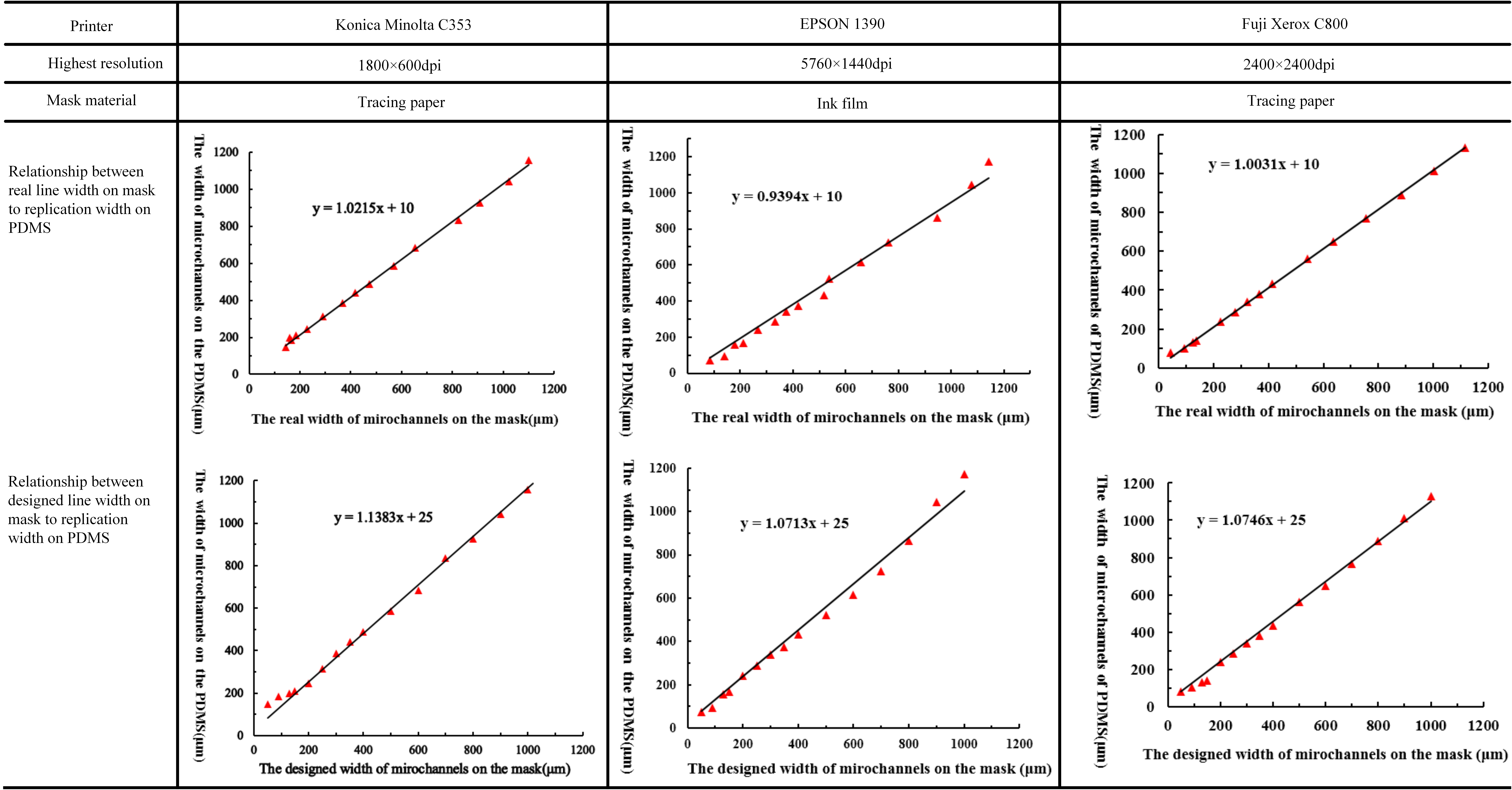


Fig.S2 Fabrication resolution of FFSL. Three typical different printers (Konica Minolta C353, Epson 1390 and Fuji Xerox C800) were used to print the mask film. Then a serial FFS molds were fabricated and micro structures based on the FFS molds were replicated with PDMS.
